# Aperiodic brain activity changes in patients with stroke following virtual reality-based upper limb robotic rehabilitation: a pilot Randomized Controlled Trial

**DOI:** 10.3389/fnhum.2025.1671804

**Published:** 2025-10-17

**Authors:** Maria Cristina Mauro, Alessio Fasano, Marco Germanotta, Laura Cortellini, Sabina Insalaco, Arianna Pavan, Angela Comanducci, Eugenio Guglielmelli, Irene Giovanna Aprile

**Affiliations:** ^1^IRCCS Fondazione Don Carlo Gnocchi ONLUS, Florence, Italy; ^2^IRCCS Fondazione Don Carlo Gnocchi ONLUS, Milan, Italy; ^3^Department of Engineering, Università Campus Bio-Medico di Roma, Rome, Italy

**Keywords:** robotics, rehabilitation, quantitative electroencephalography (QEEG), virtual reality (VR), Spectral Exponent Index (SEI)

## Abstract

**Introduction:**

Stroke-related brain changes have traditionally been studied through oscillatory electroencephalographic (EEG) activity, but recent evidence highlights the value of aperiodic components. This pilot randomized controlled trial aimed to assess stroke-related aperiodic EEG changes following virtual reality-based robotic rehabilitation using the Spectral Exponent Index (SEI).

**Methods:**

Nineteen patients with subacute stroke were randomized to unilateral (*n* = 9) or bilateral (*n* = 10) upper limb training with a robotic exoskeleton (30 sessions). EEG was recorded at rest before (T0), after (T1), and at 1-week follow-up (T2). SEI was computed for hemispheric and sensorimotor clusters, in affected (AH) and unaffected (UH) hemispheres. Clinical evaluation was performed at T0 and T1 with validated clinical scales.

**Results:**

At T0, the SEI in the sensorimotor cluster of the AH was significantly lower than in the UH. At T1, the SEI in the AH increased together with clinical improvements in upper limb motor function. At T2, the SEI in the AH decreased again and was lower than in the UH. No differences were found between unilateral and bilateral groups.

**Discussion:**

Robotic rehabilitation modulated the aperiodic EEG background in the affected hemisphere of patients with stroke, particularly in sensorimotor areas. These SEI changes mirrored motor recovery, suggesting that it may represent a useful biomarker to track localized neural mechanisms of functional improvement after stroke. No differences between unilateral and bilateral training likely reflect the pilot sample size or shared cortical mechanisms of action activated by both rehabilitation approaches.

**Clinical Trial Registration:**

ClinicalTrials.gov registration number: NCT05176600.

## 1 Introduction

Stroke is the second leading cause of mortality and the primary cause of disability in adults worldwide, responsible for approximately 11% of total deaths in 2019, as reported by the World Health Organization (WHO) (Katan and Luft, 2018). Neurological impairments following a stroke affect sensory perception, sensory motor integration, motion and force production, leaving 15% to 30% of stroke survivors severely disabled (Roger et al., 2011). In particular, stroke induces significant motor deficits of the upper limb (UL), leading to a loss of independence and a substantial reduction in the quality of life (Wade et al., 1983).

Many therapeutic approaches have been put forward in rehabilitation settings to promote functional recovery after stroke. In recent years, robot-assisted therapy has emerged as one of the most important technological innovations in stroke rehabilitation, providing intensive, repetitive, and task-specific practice while allowing precise measurement and standardization of training (Masiero et al., 2014; Pavan et al., 2024). Robot-based systems have the advantage of allowing programmable movement patterns, control of movement repetitions, and real-time position and force measurement (Frolov et al., 2018). Robot-assisted arm training, in particular, has shown effectiveness in improving motor functions and daily living independence (Aprile et al., 2020; Tekin et al., 2025; Verola et al., 2025). Advances in upper-limb exoskeletons now enable customized trajectories, force feedback, and bilateral training paradigms, which can be seamlessly combined with virtual reality (VR) environments (Gueye et al., 2021). VR can enhance neuroplasticity and recovery after a stroke by providing more intensive and engaging training (Kim et al., 2020). This is possible due to several benefits, among which offering rehabilitation tasks with different levels of difficulty, providing immediate feedback that enhances the training, creating more immersive and engaging experiences, ensuring a more consistent approach to rehabilitation, and allowing for safe simulation of real-life daily activities (Kim et al., 2020). In particular, combined robotic training and VR programs for UL stroke rehabilitation recently showed favorable effects on motor outcomes and activities of daily living (Mani Bharathi et al., 2024; Alashram, 2024).

Among mechanisms sustaining clinical recovery after a stroke, cerebral or synaptic plasticity, defined as the neural ability to change brain functional organization over time producing different responses to the same stimulus, seems to play a crucial role (Tecchio et al., 2006). In this regard, the monitoring of electrical brain activity with electroencephalography (EEG) could be an accessible and versatile instrument to aid the care of patients with stroke (Lanzone et al., 2022).

In literature, several quantitative EEG (qEEG) indices have been proposed to capture modifications in brain activity following stroke both in the acute and chronic phases (Lanzone et al., 2023). Traditionally, qEEG metrics have focused on oscillatory activity in different brain waves, therefore analyzing the periodic components in the different frequency bands. Trujillo et al. (2017) found that the Power Ratio Index, an index measuring the relationship between the power bands, was significantly correlated with the Fugl-Meyer assessment after unilateral robotic rehabilitation in patients with chronic stroke.

Unilateral robotic training delivers intensive, task-specific practice to the affected limb, which may enhance local cortical reorganization in the lesioned hemisphere. By contrast, bilateral robotic training engages both limbs simultaneously, promoting interhemispheric communication and functional connectivity across sensorimotor cortices, a process associated with more balanced cortical excitability and facilitation of bimanual coordination (Tang et al., 2023). Hence, comparing these approaches is essential to discern whether recovery is primarily driven by focal reorganization or by broader interhemispheric coupling.

We have recently found that bilateral robotic rehabilitation after stroke yields, along with a clinical improvement in the upper extremity function, a restoration of interhemispheric activity balance measured with the pairwise-derived Brain Symmetry Index (pdBSI), an index of symmetry of cortical oscillation activities between hemispheres (Mauro et al., 2024). While this study provided valuable insights into the interhemispheric balance, it did not address potential changes in the background dynamics of cortical oscillations.

Recent findings emphasize the significance of the aperiodic (power-law) structure in characterizing pathological brain states (Lanzone et al., 2022). In particular, a 1/f-like shape is a general property of the brain activity (He et al., 2010), indicating that the “background” of the EEG power spectrum decays from slower to faster frequencies. Patients with stroke exhibit a remarkable temporal slowing of the EEG, i.e., a steeper decay, typically described by an increase in delta/alpha ratio (Finnigan et al., 2016). Lanzone et al. (2022) proposed instead the use of the Spectral Exponent Index (SEI), a metric that reflects EEG slowing by quantifying the power-law decay of the EEG Power Spectral Density (PSD). Compared to the pdBSI, which measures global interhemispheric symmetry in oscillatory EEG activity, the SEI captures changes in the aperiodic component of the EEG power spectrum, potentially providing a more sensitive marker of neuroplastic changes during rehabilitation. Lanzone et al. assessed the sensitivity of the SEI to the effects of both acute and chronic stroke, and its modulation following 1 month of traditional physical rehabilitation. They showed that the SEI is a reliable marker of the neurophysiological alterations occurring after stroke, capable of identifying the lesioned hemisphere, and tracking the clinical recovery after traditional physical rehabilitation. Johnston et al. (2023) found that in patients with chronic stroke, the abnormal steepening of the aperiodic spectral components in resting state magnetoencephalographic activity could be detected even over the unlesioned hemisphere, though it was most pronounced in perilesional areas. However, no previous study has analyzed the effects of VR-based robotic rehabilitation for the upper limb on the EEG-derived SEI in patients with stroke.

Building on our previous findings (Mauro et al., 2024), the aim of the present study is to implement the SEI to evaluate aperiodic brain changes elicited by robot-assisted rehabilitation using VR in patients with subacute stroke undergoing bilateral or unilateral upper limb treatment through a bilateral upper limb exoskeleton. Our hypothesis is that upper limb robotic rehabilitation, whether unilateral or bilateral, would lead to a reduction in EEG slowing, reflected by a less negative SEI after the treatment program. We also expect this renormalization of the SEI to be more prominent in the affected (lesioned) hemisphere than the unaffected one, in the sensorimotor cortical regions. Accordingly, we expect SEI changes to mirror clinical improvements in upper limb motor outcomes.

## 2 Materials and methods

### 2.1 Study design and participants

We enrolled consecutive subjects with stroke, verified by Magnetic Resonance Imaging (MRI) or Computed Tomography (CT). Patients were enrolled at the Santa Maria della Provvidenza Centre of Fondazione Don Carlo Gnocchi ONLUS, in Rome (Italy) between January 2022 and November 2022. The eligibility criteria were: (1) age between 18 and 85 years; (2) ischemic stroke; (3) first cortical and supratentorial event; (4) moderate upper extremity motor deficit (evaluated by Fugl-Meyer Assessment for Upper Extremity score between 29 and 42); (5) time since the acute event between 1 month and 6 months; (6) Trunk Control Test score greater than or equal to 48. Exclusion criteria were: (1) significant medical comorbidity (such as severe neurological disease, cardiovascular disease, diabetes/unstable hypertension); (2) cognitive impairment that prevents comprehension of commands and administered exercises; (3) inability or unwillingness to provide informed consent. All participants gave their written informed consent according to the Declaration of Helsinki. This is a secondary analysis of a pilot randomized controlled trial (RCT), designed to compare the effects of unilateral vs. bilateral upper limb rehabilitation using a bilateral upper limb exoskeleton in patients with subacute stroke (Mauro et al., 2024). The trial was approved by the Ethics Committee of Lazio 1 (609/CE Lazio 1) and registered at ClinicalTrials.gov with identifier number NCT05176600.

### 2.2 Intervention

Subjects who met the criteria were enrolled in a 30-session program for upper-limb neurorehabilitation, using the Arm Light Exoskeleton Rehab Station (ALEx RS, Wearable Robotics Srl). Each rehabilitation session lasted 45 min, with a frequency of five times a week. Each patient was randomly assigned to either the unilateral treatment group (UG) or the bilateral treatment group (BG).

In the UG, patients utilized the ALEx RS robot in its unilateral configuration to perform upper limb movements with the affected arm, accompanied by the relevant VR exergames. Conversely, in the BG, the ALEx RS robot was employed in its bilateral configuration alongside the corresponding VR exergames. Both groups performed rehabilitation treatments in the presence of a physical therapist and an engineer. Details about unilateral and bilateral treatments are reported in our previous paper (Mauro et al., 2024).

In addition to the UL robotic rehabilitation session according to the allocated group, all subjects underwent conventional rehabilitation sessions (six times/week), lasting 45 min, focused on lower limbs, sitting and standing training, balance, and walking. Subjects also underwent occupational and speech therapy, if needed.

The randomization sequence was generated by using the R (version 3.3.0, R Core Team, Vienna, Austria) package blockrand, with random block sizes ranging from 2 to 8. Randomization was stratified according to age (younger, <65 years; older, ≥65 years), to ensure that the subjects' numbers and characteristics in each group were closely matched. The randomization list was prepared by an investigator with no clinical role in the study. Due to the nature of the interventions, it was not possible to blind participants or treating therapists to treatment allocation.

### 2.3 Robotic device

**Figure 1:**
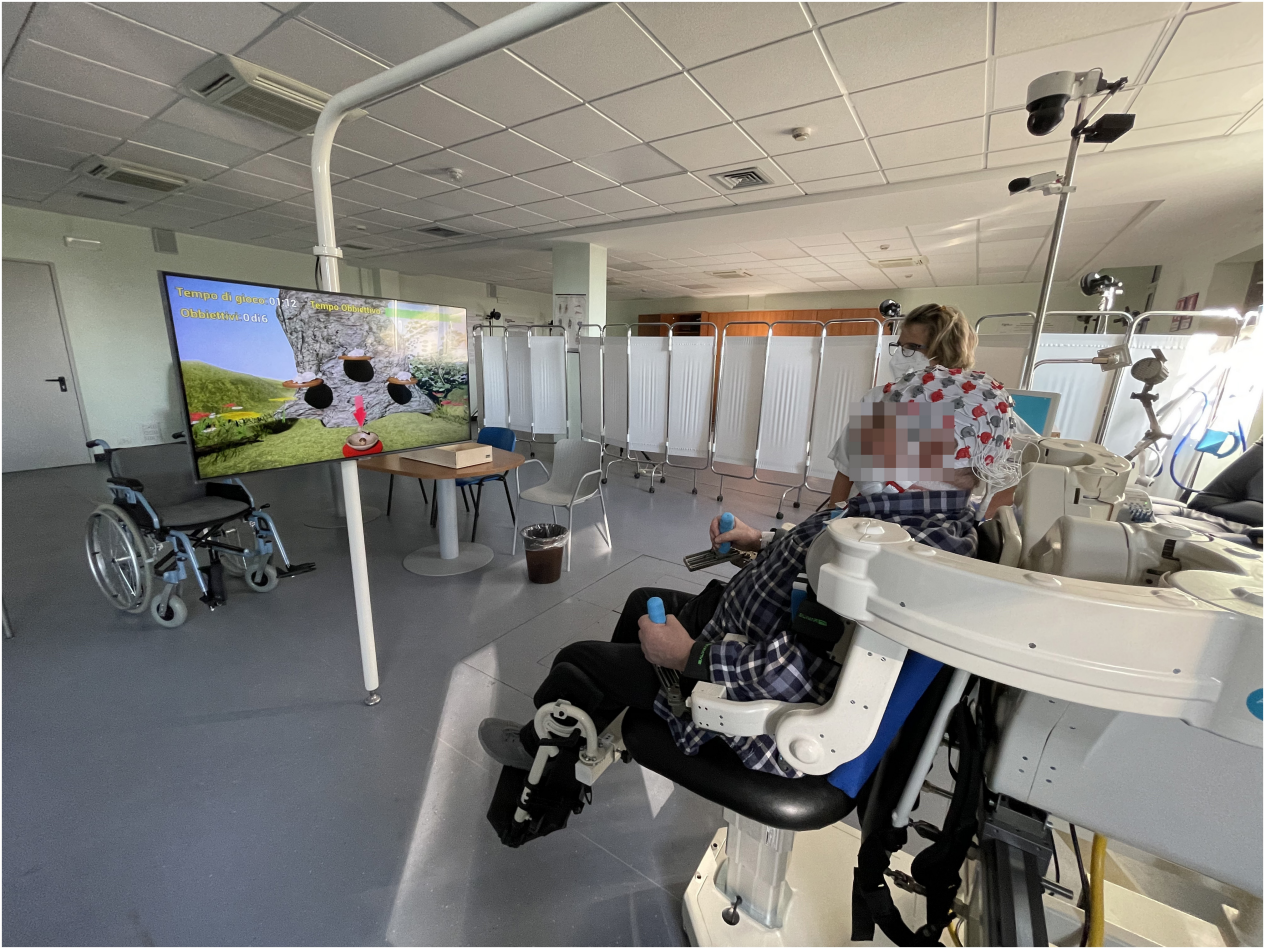
Arm light exoskeleton rehab station (ALEx RS).

The robotic sessions were carried out using the UL exoskeleton ALEx RS (Figure 1), which consists of two independent and symmetrical exoskeletons, one for the right UL and the other for the left UL, each with six degrees of freedom (four actuated and sensorized, and two sensorized). Depending on the treatment being administered to the patient, the two exoskeletons can be used concurrently (bilateral configuration) or separately (unilateral configuration). The exoskeleton handles are sensorized and can measure movement. The robot is provided of an automatic support system that assists the patient in reaching the target in case he/she is unable to perform or complete the movement in a given time.

The device includes several exergames in VR that can be selected by the operator based on the patient's needs. The exercises are of various types and are intended to stimulate the patient's concentration, allowing the patient to carry out cognitive as well as motor rehabilitation. Exercises, for both configurations, involve 3D reaching (on the frontal and sagittal plane), picking and lifting objects, trajectory tracking and involved proprioception and visuomotor abilities, coordination specifically targeting visual-spatial exploration, oculo-manual coordination, reflex speed, association, concentration and attention. In addition, the bilateral configuration focuses also on enhancing upper limbs cooperation ability, thereby improving coordination and procedural memory in performing bimanual tasks.

### 2.4 Clinical assessment

Clinical evaluations were performed by a physical therapist at baseline (T0) and after 30 sessions of treatment (T1) using the following scales: (i) the Fugl-Meyer Assessment for upper extremity (FMA-UE) (Fugl-Meyer et al., 1975); (ii) the Action Research Arm Test (ARAT) (Lyle, 1981); (iii) the Motricity Index (MI) (Bohannon, 1999); (iv) the Modified Ashworth Scale (MAS) (Bohannon and Smith, 1987); (v) the Wolf Motor Function Test (WMFT) (Wolf et al., 2001). The evaluators who performed the clinical assessment were blinded to the treatment assignment. Details about clinical assessment are reported in our previous paper (Mauro et al., 2024).

### 2.5 EEG assessment

All subjects underwent high-density EEG (HD-EEG) recordings. The EEG data were collected with a 64-channel HD-EEG system (HD TruScan EEG; DEYMED Diagnostic) with a sampling frequency of 3 kHz. The signals were acquired using a cap with 64 Ag/AgCl scalp monopolar electrodes placed according to the International 10/20 montage. Contact impedance was kept under 5KΩ. Data was exported to EDF format for further analysis.

Recordings were acquired at the following time points: before (T0), and right after (T0+) the beginning of the first rehabilitation session, the day after the end of 30 treatment sessions (T1), and 1-week follow-up (T2).

The neurophysiological evaluation performed immediately after the start of the first rehabilitation session (T0+) aimed to assess the short-term effects of the intervention, specifically exploring potential immediate brain modifications associated with motor recovery. Additionally, the neurophysiological assessment conducted at the 1-week follow-up (T2) sought to determine whether these neural changes persisted over a short-term period of 1 week.

Resting state EEG recordings were conducted for 10 min with eyes open and 10 min with eyes closed, while the subject was relaxed and in a comfortable supine position. The assessors who performed the EEG assessment were blinded to the treatment assignment.

### 2.6 EEG signal processing

Signal processing and analyses were performed offline using MATLAB (Mathworks, Natick, MA, USA) with custom scripts. Sampled EEG data were imported into the software from EDF format with acquisition reference, and additional information regarding the channel location was added to the EEG structure. Single bad channels were removed by visual inspection and successively interpolated (nearest neighbor). Data were resampled at 1 kHz and filtered with an IIR high-pass (5th order Butterworth filter with a 0.5 Hz cut-off) and a notch filter centered at 50 Hz. Data were then re-referenced to average reference. Independent Component Analysis (ICA) was performed using the logistic infomax ICA algorithm, implemented in EEGLAB, to discriminate non-cerebral signal sources. The ICA decomposition was guided by automated rejection methods and supervised by an expert user via visual inspection. Indeed, only components with clear ocular and muscle artifacts were rejected by visual inspection of the component's topography, time-frequency, and time series. On average, three–six ICA components per recording were rejected, primarily corresponding to ocular and muscle artifacts. Power Spectral Density (PSD) was computed using Welch's method (2 s window, 50% overlap). The eyes-closed data were carefully inspected to minimize the influence of residual alpha peaks.

Signal analyses were performed by an investigator with no clinical role in the study and blinded to the randomization groups.

### 2.7 Spectral Exponent Index

Subsequently, the Spectral Exponent Index (SEI) was estimated in the 1–20 Hz frequency range, covering the key spectral bands involved in post-stroke alterations (Johnston et al., 2023; Ajčević et al., 2021), using a custom-made MATLAB code for four different clusters of channels: two hemispheric clusters (one for the left and one for the right hemisphere) and two subclusters covering the sensorimotor area (one for the left and one for the right hemisphere). The hemispheric clusters include the following channels: (i) F6, FC6, C6, CP6, P6, F4, FC4, C4, CP4, P4, F2, FC2, C2, CP2, P2 (for the right hemisphere); (ii) F5, FC5, C5, CP5, P5, F3, FC3, C3, CP3, P3, F1, FC1, C1, CP1, P1 (for the left hemisphere). The sensorimotor clusters include the following channels: (i) C4, CP4, P4, C2, CP2, P2 (for the right hemisphere); (ii) C3, CP3, P3, C1, CP1, P1 (for the left hemisphere).

The SEI quantifies the steepness of the decay of the EEG PSD background. The PSD background refers only to the aperiodic component of the PSD once the bias due to oscillatory peaks was minimized by smoothing them. The PSD background (i.e., non-oscillatory component) decays, from lower (hence slower) to higher (hence faster) frequencies, approximately according to an inverse power-law (Pritchard, 1992):


(1) $$\begin{array}{l} \text{PSD}\left(\text{f}\right)=\;\frac{1}{{\text{f}}^{\alpha }} \end{array}$$

So, the SEI was computed for each pre-processed EEG channel, as follows:


(2) $$\begin{array}{l} \text{SEI }=\;\beta =\;-\;\alpha \end{array}$$

where β is the SEI as defined by Colombo et al. (2019), and α is the slope of the decay of the PSD background. Specifically, the three-step procedure of Colombo et al. (2019) was followed in order to estimate the spectral exponent β of the background PSD:

1. fitting of a first ordinary least-square (OLS) line to the PSD.
2. frequency bins with positive residuals larger than 1 median absolute deviation of the residual distribution were discarded, as likely containing oscillatory peaks. Adjacent bins with positive residuals were also discarded, so as to remove both the top and the base of the peak.
3. a second ordinary least-square (OLS) line was then fit on the remaining frequency bins (without oscillatory peaks). The slope of this second line was considered as the spectral exponent β of the PSD background.

In order to obtain a single estimate of the spectral exponent across the scalp, the average SEI across electrodes, in the sensorimotor or hemispheric clusters, was considered for each participant.

### 2.8 Statistical analysis

The statistical analysis was performed to evaluate the changes in the neurophysiological and clinical data induced by the robotic rehabilitation treatment.

Normal data distribution was confirmed by Shapiro–Wilk test.

To compare SEI values at T0 between treatment groups, independent samples Student's *t*-test was conducted.

To compare neurophysiological data between groups, and to assess changes in SEI over time and across hemispheres, a mixed-design Analysis of Variance (ANOVA) was performed. Time (T0, T1, T2) and Hemisphere (affected or AH, unaffected or UH) served as within-subject factors, while Group (unilateral, bilateral) was treated as a between-subject factor.

Moreover, an explorative analysis was conducted to evaluate the short-term effects of treatment, i.e., after one 45 min-session, by means of a mixed ANOVA test performed considering Time (two levels: T0 vs. T0+) and Hemisphere (two levels: AH vs. UH) as within-group factors, and Group (two levels: unilateral vs. bilateral) as a between-group factor.

For clarity, in the ANOVA outputs, the label “interaction” refers to the statistical interaction between the within-subject factor, e.g., Hemisphere (affected vs. unaffected), and other factors (e.g., Time, Group). For instance, it indicates whether SEI changes differ between the two hemispheres across sessions or rehabilitation groups, rather than representing a separate neurophysiological measure.

Mauchly's test was used to confirm sphericity, while homoscedasticity was assessed through the Levene's test. Greenhouse-Geisser corrections were applied to account for potential violations of sphericity hypothesis.

*Post-hoc* pairwise comparisons, with *p*-values adjusted for multiple comparisons through Šidák correction, were performed to explore significant interactions and main effects. The same analysis was repeated two times: one considering data from the hemispheric clusters, another considering only data from the sensorimotor clusters, in order to assess the sensitivity of the SEI in detecting local variations in “temporal slowing” specifically within the cortical areas impacted by stroke-related damage, as opposed to broader, global cortical effects.

For all the analyses, we evaluated only data acquired in the eyes closed condition in order to minimize artifacts due to eye and scalp muscle movements with respect to the eyes open condition, and considering that Colombo et al. (2019) observed that the SEI is only slightly influenced by local spectral peaks (e.g., the alpha peak).

With respect to clinical data, a mixed ANOVA test was conducted, considering Time (two levels: T0 vs. T1) as a within-group factor, and Group (2 levels: unilateral vs. bilateral) as a between-group factor.

A *p*-value lower than 0.05 was considered as significant. Statistical analysis was performed using the SPSS Statistics software (version 28, IBM Corp., Armonk, NY, USA), by an investigator with no clinical role in the study and blinded to the randomization groups.

## 3 Results

### 3.1 Sample

**Figure 2:**
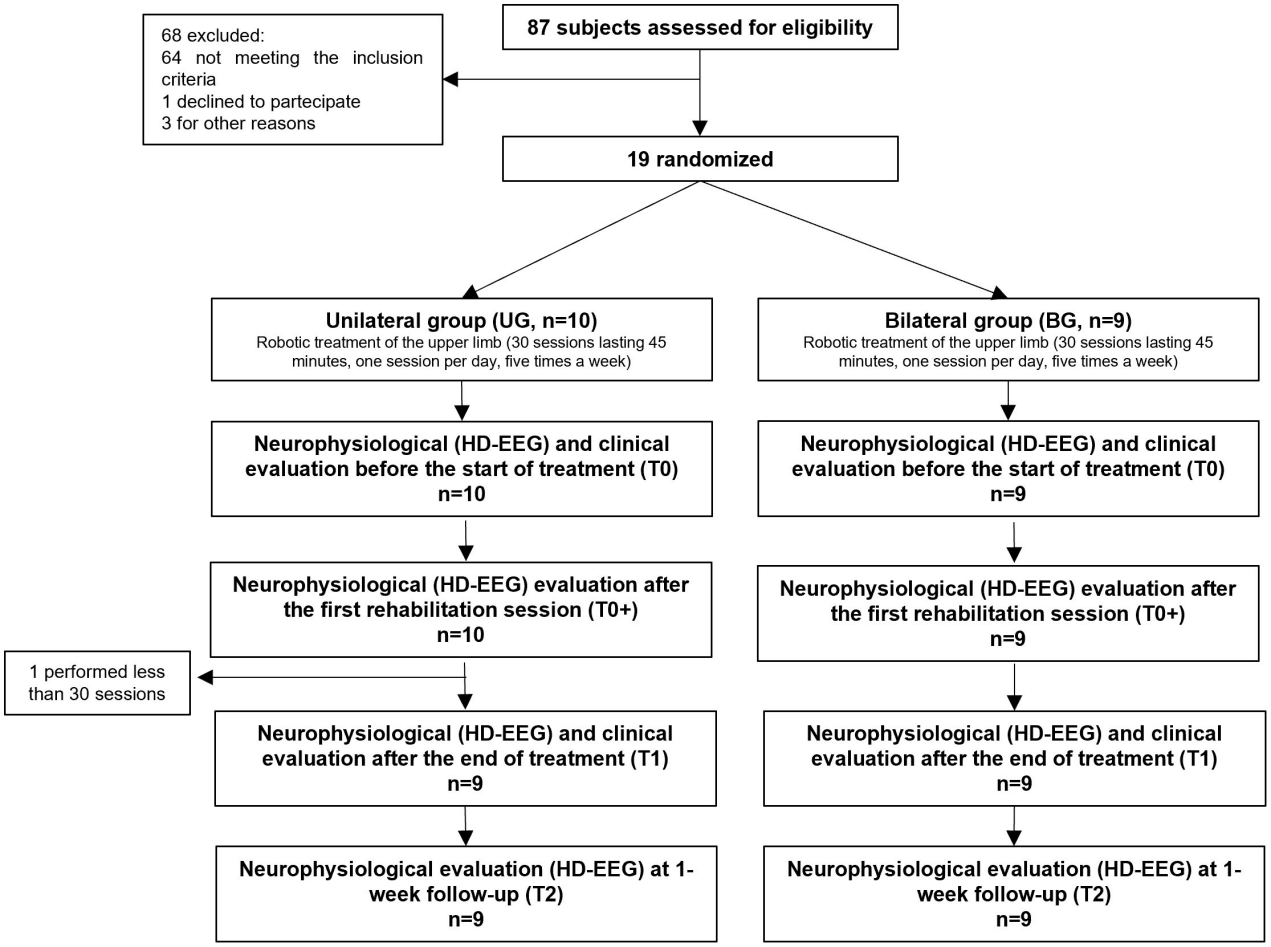
CONSORT flow diagram of participant screening, allocation, treatment, evaluations and follow-up. Total number of participants (*n*) are shown at each stage.

A flowchart of the trial stages (CONSORT diagram), with patients' screening, allocation, treatment, evaluations, and follow-up is presented in Figure 2. We have used the same sample of our previous study (Mauro et al., 2024), which we report here for clarity. A total of 87 patients were screened for eligibility. Among these, 64 were excluded based on the inclusion criteria, one declined participation and three were excluded for other reasons unrelated to the study. Nineteen patients, who met the inclusion criteria and provided informed consent, were then randomized into the UG (*n* = 10) or BG (*n* = 9) treatment groups. Of those, one participant from the UG completed fewer than 30 treatment sessions due to reasons unrelated to the study and did not undergo evaluations at T1 and T2. Consequently, 18 patients (UG, *n* = 9; BG, *n* = 9) were assessed at T1 and T2 and included in the analysis.

The baseline characteristics of the two treatment groups (UG vs. BG) are summarized in Table 1. The baseline values were compared between groups by means of Mann–Whitney *U*-test and Chi-squared test, respectively for numeric and categorical variables. The two groups were comparable in terms of age, sex, index stroke location, affected side, dominant side, time from onset to randomization, and clinical scales at baseline. Assumption checks confirmed that the ANOVA requirements were met. A Shapiro–Wilk test on the model residuals showed no departure from normality (*W* = 0.977, *p* = 0.383). Levene's test confirmed homogeneity of variance for SEI between treatment groups [*F*_(1, 53)_ = 0.351, *p* = 0.556], and separate Levene tests at each timepoint also indicated equal variances (T0: *p* = 0.892; T2: *p* = 0.134; T3: *p* = 0.650). Mauchly's test (*W* = 0.803, *p* = 0.193) indicated that the assumption of sphericity has been met for all the data samples.

**Table 1:** Baseline characteristic of the sample.

**Characteristics**	**Unilateral group (*n* = 10)**	**Bilateral group (*n* = 9)**	***p*-value**
Age, years	68.9 (14.7)	70.2 (4.9)	0.447
**Sex**			
Men	6 (60%)	4 (44.4%)	0.498
Women	4 (40%)	5 (55.5%)	
**Index stroke location (ischemic stroke)**			
Lacunar stroke	0 (0.0%)	1 (11.1%)	0.449
Partial anterior circulation stroke	8 (80%)	5 (55.5%)	
Total anterior circulation stroke	2 (20%)	2 (22.2%)	
Posterior circulation stroke	0 (0.0%)	1 (11.1%)	
**Affected side**			
Right	6 (60%)	6 (66.6%)	0.764
Left	4 (40%)	3 (33.3%)	
**Dominant side**			
Right	9 (90%)	9 (100%)	0.329
Left	1 (10%)	0 (0%)	
Days from index stroke to enrolment	107.6 (49.3)	93.5 (42.6)	0.604
Fugl-Meyer upper extremity motor function score (0–66)	35.2 (22.6)	36.3 (13.9)	1.000
Fugl-Meyer sensory function	7.7 (3.9)	8.4 (3.9)	
Motricity Index Upper Limb (0–100)	56.2 (31.1)	64.6 (16.7)	0.604
**Modified Ashworth Scale (0–4)**			
Shoulder abduction	0.6 (1.0)	0.6 (0.7)	0.842
Shoulder intrarotation	0.8 (1.0)	0.7 (0.7)	1.000
Elbow	1.2 (0.9)	1.2 (0.8)	0.968
Wrist	0.7 (0.8)	0.8 (0.6)	0.720
Action research arm test (0–45)	24 (24)	25 (23)	0.905
Wolf motor function test	37 (32)	41 (22)	0.968

Data are mean (SD) or N (%). *p*-values refer to Mann–Whitney U-test and chi-squared.

### 3.2 Clinical assessment

**Figure 3:**
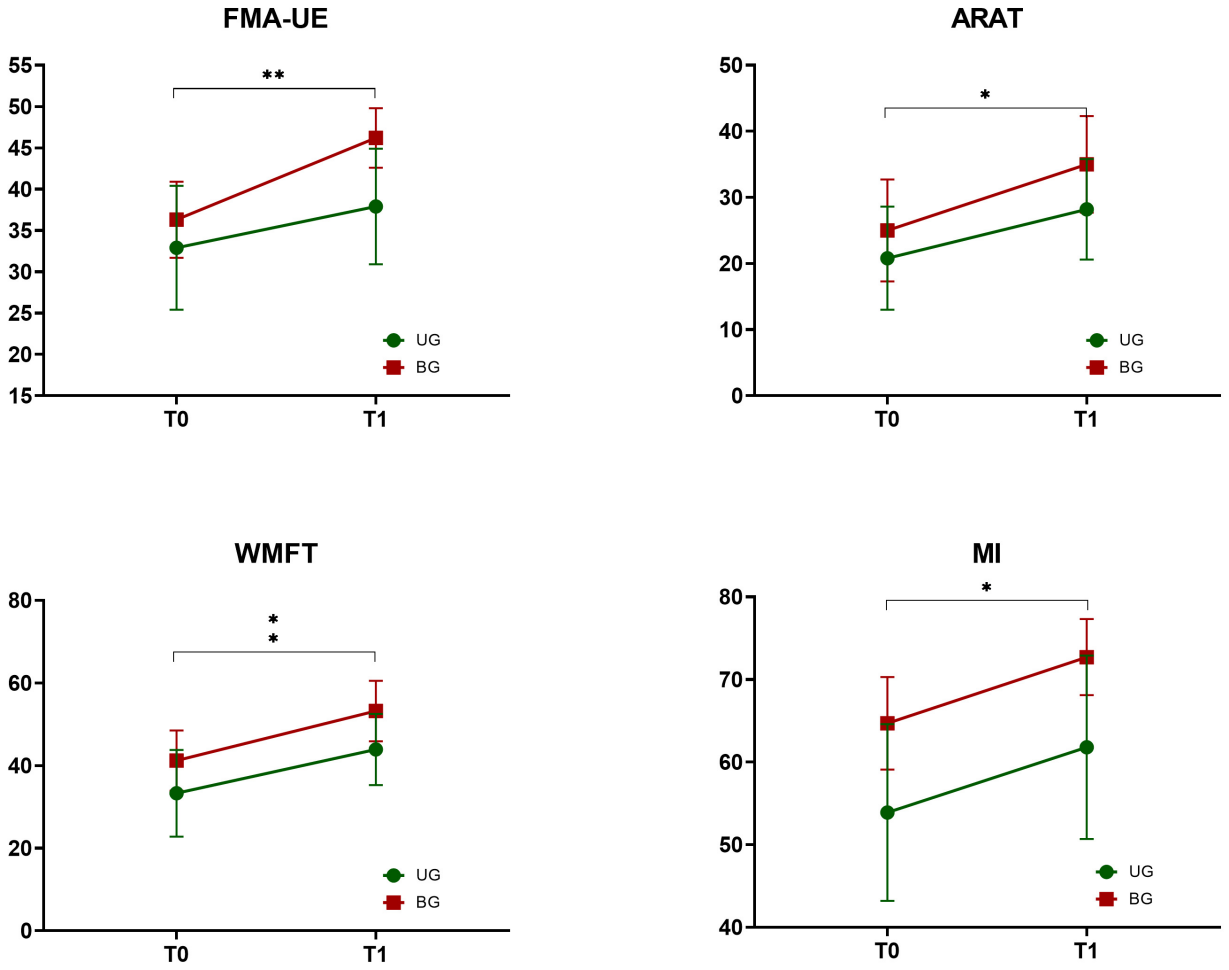
Clinical scales before (T0) and after (T1) rehabilitation, for the unilateral (UG, green) and bilateral (BG, red) group. FMA-UE, Fugl-Meyer assessment for upper extremity; ARAT, action research arm test; WMFT, wolf motor function test; MI, Motricity Index. *p < 0.05, **p < 0.01.

Figure 3 shows the mixed ANOVA results related to clinical evaluation. As reported in detail in our previous work (Mauro et al., 2024), no significant interaction between time and group was found. With respect to the main effect time, it was statistically significant in the following clinical scales: FMA-UE motor function (*p* = 0.001), ARAT (*p* = 0.015), WMFT (*p* = 0.007), and MI (*p* = 0.025). In contrast, it was not statistically significant for the MAS (shoulder *p* = 0.415; elbow *p* = 0.867; wrist *p* = 0.412) and the FMA-UE sensation (*p* = 0.471).

### 3.3 Neurophysiological evaluation

The independent samples Student's *t*-test showed that the SEI values at baseline (T0) were not significantly different between UG and BG in the hemispheric and affected cluster (*p* = 0.851), hemispheric and unaffected cluster (*p* = 0.628), sensorimotor and affected cluster (*p* = 0.885), sensorimotor and unaffected cluster (*p* = 0.469).

The mixed-design ANOVA revealed distinct patterns in SEI variations when analyzing hemispheric and sensorimotor clusters separately.

### 3.4 Hemispheric cluster analysis

**Figure 4:**
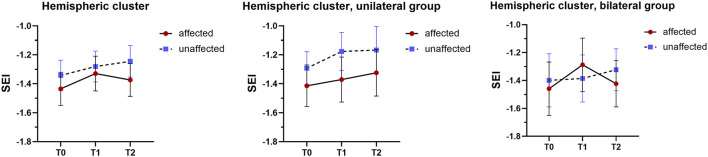
Spectral Exponent Index (SEI) in the hemispheric cluster for all patients **(left)**, for the unilateral group **(center)**, and for the bilateral group **(right)**. SEI values are averaged over the hemispheric cluster, i.e., all electrodes of each hemisphere (affected: red; unaffected: blue). T0: baseline timepoint; T1: end of rehabilitation; T2: follow-up.

Figure 4 shows the SEI in the hemispheric cluster, in the AH and UH, for the whole sample and for unilateral and bilateral groups. No significant Time × Group, Time × Hemisphere, Hemisphere × Group, nor Time × Hemisphere × Group interaction effects were observed. A significant effect of Hemisphere factor [*F*_(1, 16)_ = 5.873, *p* = 0.028, η^2^ = 0.268] was found, with the AH (−1.380 ± 0.113) displaying significantly lower SEI than the UH (−1.284 ± 0.106), independently of time and group (see Figure 4, left panel).

In the explorative analysis (Table 2), performed to investigate the neurophysiological short-term effect of the robotic treatment, i.e., right after the first rehabilitation session, a significant main effect of Hemisphere was observed, indicating differences between the affected and unaffected hemispheres [*F*_(1, 17)_ = 4.555, *p* = 0.048, partial η^2^ = 0.211] regardless of time and group, in particular being the mean SEI in the AH (−1.432 ± 0.121) significantly lower than the mean SEI in the UH (−1.347 ± 0.114).

**Table 2:** Spectral Exponent Index (SEI) in the hemispheric clusters (affected hemisphere, AH, and unaffected hemisphere, UH), at enrolment (T0) and at the end of the first session (T0+) for the two groups, separately, along with the results of statistical analysis (*p*-values for the main effects of Hemisphere, Time and Group, and their interactions).

**Hem**	**Group**	**T0 (mean ±SE)**	**T0+ (mean ±SE)**	**Main effects (** ***p*** **-value)**			**Interactions (** ***p*** **-value)**			
				**Time**	**Group**	**Hem**	**HemXTime**	**TimeXGroup**	**HemXGroup**	**HemXTimeXGroup**
AH	Bi	−1.459 ± 0.171	−1.473 ± 0.182	0.939	0.619	0.048	0.694	0.271	0.234	0.192
	Uni	−1.414 ± 0.162	−1.383 ± 0.173							
UH	Bi	−1.399 ± 0.157	−1.461 ± 0.176							
	Uni	−1.292 ± 0.149	−1.235 ± 0.170							

bi, bilateral; uni, unilateral; Hem, Hemisphere factor. Interaction terms: Hem × Time, interaction between Hemisphere (AH vs. UH) and Time (T0 vs. T0+); Time × Group, interaction between Time and Group (UNI vs. BI); Hem × Group, interaction between Hemisphere and Group; Hem × Time × Group, three-way interaction among Hemisphere, Time, and Group.

### 3.5 Sensorimotor cluster analysis

**Figure 5:**
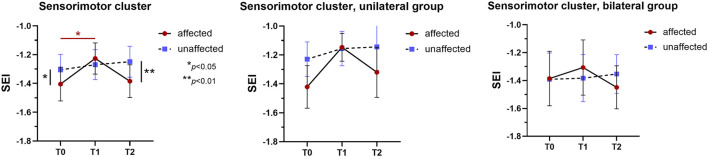
Spectral Exponent Index (SEI) in the sensorimotor cluster for all patients **(left)**, for the unilateral group **(center)**, and for the bilateral group **(right)**. *p < 0.05, **p < 0.01. The red colored asterisk refers to the significant difference between SEI in the affected sensorimotor cluster at timepoint T0 (before rehabilitation) and the one at timepoint T1 (after rehabilitation). The black colored asterisks refer instead to the comparison between affected and unaffected hemispheres at timepoints T0 and T2 (follow-up). SEI values are averaged over the sensorimotor cluster, i.e., electrodes anatomically over primary motor and somatosensory cortex, for each hemisphere (affected: red; unaffected: blue).

Figure 5 shows the SEI in the sensorimotor cluster, in the AH and UH, for the whole sample and for unilateral and bilateral groups. The results showed no significant Time × Group, Hemisphere × Group, nor Time × Hemisphere × Group interaction effects, but a significant Time × Hemisphere interaction [*F*_(2, 15)_ = 5.208, *p* = 0.027 Greenhouse-Geisser corrected, η^2^ = 0.246]. Specifically, *post-hoc* pairwise comparisons showed that in the AH, SEI at T1 was significantly higher than SEI at T0 (mean difference T0 – T1 = −0.172, *p*-adj = 0.037), regardless of the group (bilateral or unilateral). Additionally, SEI differed significantly between hemispheres at both T0 (mean difference AH – UH = −0.107, *p*-adj = 0.031) and T2 (mean difference AH – UH = −0.136, *p*-adj = 0.009).

In the explorative analysis on T0+ (Table 3), no significant effect of Time, Hemisphere, Group, nor of their interactions was observed.

**Table 3:** Spectral Exponent Index (SEI) in the sensorimotor clusters (affected hemisphere, AH, and unaffected hemisphere, UH), at enrolment (T0) and at the end of the first session (T0+) for the two groups, separately, along with the results of statistical analysis (*p*-values for the main effects of Hemisphere, Time and Group, and their interactions).

**Hem**	**Group**	**T0 (mean ±SE)**	**T0+ (mean ±SE)**	**Main effects (** ***p*** **-value)**			**Interactions (** ***p*** **-value)**			
				**Time**	**Group**	**Hem**	**HemXTime**	**TimeXGroup**	**HemXGroup**	**HemXTimeXGroup**
AH	Bi	−1.386 ± 0.176	−1.423 ± 0.184	0.916	0.664	0.111	0.675	0.285	0.095	0.634
	Uni	−1.421 ± 0.167	−1.359 ± 0.174							
UH	Bi	−1.391 ± 0.159	−1.427 ± 0.189							
	Uni	−1.229 ± 0.149	−1.202 ± 0.179							

bi, bilateral; uni, unilateral; Hem, Hemisphere factor. Interaction terms: Hem × Time, interaction between Hemisphere (AH vs. UH) and Time (T0 vs. T0+); Time × Group, interaction between Time and Group (UNI vs. BI); Hem × Group, interaction between Hemisphere and Group; Hem × Time × Group, three-way interaction among Hemisphere, Time, and Group.

## 4 Discussions

In the present study, we employed a synthetic qEEG measure, the Spectral Exponent Index (SEI), to evaluate aperiodic brain changes elicited by VR-based robotic rehabilitation in patients with subacute stroke.

Previously, Lanzone et al. (2022) introduced the SEI for longitudinal assessment of recovery from stroke. In particular, the authors compared patients with ischemic stroke, undergoing 1 month of traditional physical rehabilitation, with healthy controls. Their findings indicated that, before rehabilitation, patients with stroke exhibited significantly more negative SEI values than healthy controls, which is indicative of broad-band EEG slowing. Furthermore, in patients with stroke, the SEI over the affected hemisphere was consistently more negative compared to the unaffected hemisphere and showed renormalization after the treatment.

The main contribution of our study lies in evaluating changes in SEI after a VR-based robotic rehabilitation intervention focused on the upper limb in patients with subacute stroke, comparing the bilateral vs. unilateral approach. While Lanzone et al. (2022) focused on traditional rehabilitation, our study incorporates an innovative VR-based robot-assisted rehabilitation approach focused on the upper limb. This robotic treatment, independently of unilateral or bilateral modalities, significantly improved functional recovery of the upper extremity in our patients with stroke (Mauro et al., 2024).

Notably, whereas Lanzone et al. showed SEI renormalization during the acute phase [a time-sensitive window of plasticity (Murphy and Corbett, 2009)] with conventional therapy, our subacute cohort indicates that an upper-limb VR-robotic program elicits a focal increase (i.e., less negative SEI) in the affected sensorimotor cortex at T1, which attenuates once intensive robotic practice ceases and only generalized conventional therapy continues.

Additionally, unlike Lanzone et al. (2022) using a 32-channel EEG system, our study employed HD-EEG with 64 channels to capture detailed neurophysiological changes. This advanced neurophysiological technique provides high spatial and temporal resolution, enabling a more comprehensive analysis of the brain's response to rehabilitation. This resolution allowed us to analyze changes in SEI within the sensorimotor channel cluster, i.e., the sensorimotor area. The sensorimotor area is one of the most frequently affected regions in ischemic stroke due to the vulnerability of the middle cerebral artery. The sensorimotor area of the cortex, encompassing both the primary motor cortex and the primary somatosensory cortex, is crucial for processing and executing movement as well as integrating sensory feedback. Within this region, the representation of the upper limb is particularly predominant, as evidenced by the somatotopic organization of the motor and sensory homunculi. This specialized representation is particularly evident in areas such as the precentral and postcentral gyri, where dense neuronal networks facilitate precise coordination and adaptability of upper limb functions (Bruurmijn et al., 2021).

Thus, our findings extend prior work by demonstrating that upper-limb–focused robotic therapy in the subacute phase can transiently renormalize the aperiodic background, complementing the acute-phase evidence from conventional therapy.

### 4.1 Neurophysiological improvements after rehabilitation

From a neurophysiological perspective, our findings revealed a significant increase in SEI values in the sensorimotor cluster of the affected hemisphere (AH) following the rehabilitation treatment (T1), regardless of the treatment modality (unilateral or bilateral). This suggests that the proposed intervention, independent of its bilateral or unilateral configuration, effectively promoted changes in the aperiodic structure of EEG signals, toward a reduction in the stroke-related EEG spectral slowing. Such changes are consistent with previous studies linking aperiodic EEG metrics with cortical excitability and functional recovery in patients with stroke (Lanzone et al., 2022, 2024), as well as with prior research emphasizing the role of the sensorimotor cortex in stroke recovery and its capacity for reorganization following targeted rehabilitation interventions (Ekusheva and Damulin, 2015).

Our results also indicate that the SEI improvement observed immediately post-treatment (T1) was not maintained at the 1-week follow-up (T2). A similar trend was observed in our previous findings with the pdBSI, where the initial improvement in interhemispheric symmetry at T1 was not evident at T2 (Mauro et al., 2024). This transient modulation may be interpreted as reflecting neuroplastic changes specifically induced by the stimuli provided to patients by task-specific robotic rehabilitation in a VR environment (You et al., 2005; Patel et al., 2021), consistent with early phases of a cortical reorganization, in particular a therapy-driven rebalancing of cortical excitation/inhibition within sensorimotor networks (Biskamp et al., 2022). On the one hand, the exploratory T0+ analysis revealed that a single training session did not induce measurable SEI changes, indicating that cumulative training is necessary to elicit detectable neurophysiological modulation, as suggested by rehabilitation studies (Lanzone et al., 2022, 2024). On the other hand, the lack of persistence at 1 week after treatment suggests that these modifications may require further consolidation into long-term neural adaptations, potentially mediated by structural and connectivity-level changes that SEI does not directly capture (Johnston et al., 2023; Pirovano et al., 2022; Cassidy et al., 2021; Bönstrup et al., 2019). In fact, during the follow-up period, patients continued to undergo conventional rehabilitation, but with a less specific focus on the upper limb. Therefore, the transient SEI changes may represent an early phase of cortical reorganization that triggers slower, undetectable network-level mechanisms (Pirovano et al., 2022; Cassidy et al., 2021; Bönstrup et al., 2019), which continue to support long-term functional improvements despite the apparent return of SEI to baseline levels. Future studies with longer follow-up periods and combined EEG metrics, as well as modeling dose of concomitant conventional therapy, are warranted to clarify how short-term SEI changes evolve into longer-term cortical plasticity.

### 4.2 Interhemispheric symmetry and SEI

Within the sensorimotor cluster, we found a significant difference in the SEI values between hemispheres at baseline (T0), suggesting a pre-existing imbalance in cortical activity, where the AH may exhibit decreased cortical excitability and disrupted neuroplasticity due to stroke-induced damage. This finding aligns with research indicating that stroke often leads to asymmetries in brain activity, with the affected hemisphere showing reduced functional connectivity and excitability compared to the unaffected hemisphere (Calautti and Baron, 2003). In the T0+ analysis, the hemispheric cluster revealed a significant interhemispheric difference—with the AH showing lower SEI values than the UH—that was independent of time and treatment group. This suggests that the observed difference between hemispheres is likely a pre-existing feature of the stroke (Lanzone et al., 2022) rather than an immediate effect of the first robotic treatment session. This finding underscores the importance of distinguishing baseline neurophysiological alterations from short-term treatment effects in post-stroke assessments (Donoghue et al., 2020; He, 2014).

Similarly, while at T1 the difference was reduced given the improvement of the SEI in the AH, the SEI was again lower in the AH than the UH at T2. This indicates a temporal evolution of hemispheric asymmetry of the SEI, with the index being more dynamic and sensitive to the above-mentioned neuroplasticity changes in the AH with respect to the UH. Instead, within the hemispheric cluster, the temporal patterns of the SEI were roughly similar between affected and unaffected cortical sides.

In our previous paper (Mauro et al., 2024), we found that the interhemispheric spectral symmetry, measured through the pdBSI, is restored in patients with subacute stroke following the same protocol of robotic rehabilitation used here. Notably, this result held true whether evaluated at the hemispheric level or within sensorimotor clusters. The pdBSI is indeed a global metric that provides an overarching view of hemispheric balance across brain regions. In contrast, the SEI used in the current study proved particularly valuable in identifying focal neurophysiological changes in the sensorimotor cluster of the affected hemisphere. The SEI, therefore, offers a more localized assessment, enabling us to capture neurophysiological variations directly within the cortical areas damaged and most involved in motor control and recovery. Together, these analyses contribute complementary perspectives: while the pdBSI delineates general patterns of interhemispheric reorganization, the SEI allows for the identification of focal neuroplasticity that may be masked when examining global indices. These findings underscore the value of integrating both global and localized metrics to gain a comprehensive understanding of the cortical mechanisms underlying stroke recovery and to inform the development of targeted rehabilitation strategies.

### 4.3 Combining technological rehabilitation with neurophysiological markers

The normalization of the SEI in our cohort following rehabilitation treatment came along with significant improvements in upper limb motor performance and strength. The clinical and neurophysiological improvements that we observed in our study could be due to several attributes of the device that involve the execution of tasks in VR. Recent research has found that VR-assisted exercise is beneficial in improving motor function (Chen et al., 2022), facilitating the execution of repetitive and intensive therapeutic exercises. VR can be employed to simulate real-life environments by allowing for real-time interactions and providing a means for participants to practice therapeutic, goal-oriented tasks that may not be feasible to perform in the real context due to resource constraints or safety issues. VR may also provide auditory, visual, or tactile feedback that can aid in the learning of motor skills. This type of feedback can inform individuals about their success or failure in executing therapeutic tasks, as well as motivate and encourage people to participate in rehabilitation therapy. Enhanced motivation has been associated with better concentration on therapeutic tasks, higher training intensity, and adherence to therapy (Choi et al., 2014; Levac and Sveistrup, 2014; Rohrbach et al., 2019). At the nervous system level, extensive practice can strengthen neural connections and induce reorganization in cerebral cortex regions corresponding to the affected extremity, thereby improving motor function (Colombo et al., 2019).

The observed improvements in SEI values and motor performance in our study may be attributed to the advanced capabilities of the bilateral exoskeleton and the VR environment employed here for upper limb rehabilitation. Exoskeleton devices offer precise control over (repetitive and task-specific) movement patterns, which can facilitate more effective motor learning and cortical reorganization (Gueye et al., 2021). The exoskeleton employed in our study allows for coordinated movement patterns that may facilitate interhemispheric interaction and balance, potentially leading to improvements in motor function.

This combination of precision robotic guidance and enriched VR feedback could explain why SEI increased after treatment. As discussed above, from a neurophysiological perspective, the transient modulation of SEI observed in our study may reflect an early phase of plasticity. This phase may be characterized by enhanced neural efficiency and reduction of background noise within sensorimotor networks. Indeed, the VR environment and the precision of exoskeleton-assisted training could acutely engage cortical resources, promoting a more efficient spectral profile, enhancing neural noise reduction, which is reflected in the SEI improvement. However, without continued task-specific stimulation, these changes may not consolidate into longer-term adaptations, explaining the return of SEI values toward baseline at follow-up.

Previous clinical studies have demonstrated that bilateral robotic training can enhance motor recovery and cortical reorganization (Tang et al., 2023), which are crucial for functional recovery post-stroke (McCombe Waller et al., 2014), even though the superiority of bilateral training over unilateral one has not been confirmed in some studies (Coupar et al., 2010; Dembele et al., 2024). An important result of our study is the absence of significant differences between the unilateral and bilateral groups in both clinical outcomes and SEI measures. A first explanation for this finding is the relatively small sample size of this pilot trial, which limits the statistical power to detect between-group effects (in Supplementary material we report a *post-hoc* power analysis showing low power for the group-related interaction). Another possibility is that the SEI captures neuroplasticity mechanisms that are common to both training modalities, resulting in similar renormalization of the EEG spectral decay regardless of whether the stimulation is unilateral or bilateral. This interpretation differs from our previous findings with the pdBSI, where bilateral training, but not unilateral, induced a reduction of asymmetry in delta and theta bands (Mauro et al., 2024). The discrepancy may reflect the fact that pdBSI is a global index of interhemispheric symmetry, whereas SEI is a local marker of the aperiodic background activity, less sensitive to interhemispheric dynamics. Thus, bilateral training may promote additional changes in connectivity and hemispheric balance through interhemispheric coupling, but these effects may not be captured by a slope-based measure such as SEI. Future studies with larger samples and the inclusion of connectivity-based qEEG measures will be required to better delineate modality-specific mechanisms of recovery.

Overall, our results highlight the responsiveness of the SEI to the rehabilitative intervention, supporting its use to better investigate the local neuronal mechanisms involved in stroke recovery. The increase of SEI at T1 was accompanied by significant improvements in upper-limb clinical measures, suggesting the clinical relevance of the observed neurophysiological modulation.

Indeed, the SEI demonstrated significant sensitivity and specificity in detecting neurophysiological changes post-stroke in previous studies, correlating significantly with NIHSS improvement (Lanzone et al., 2022). While the BSI and the Delta-to-Alpha Ratio have been used to assess asymmetry and oscillatory activity (Trujillo et al., 2017; Mauro et al., 2024; Liuzzi et al., 2024), the SEI provides a robust method to monitor and predict functional outcomes due to its sensitivity to broad-band EEG changes (Lanzone et al., 2022). In particular, the sensitivity of the SEI for local changes that we observed—particularly in the sensorimotor cluster of the affected hemisphere—emphasizes its potential for personalizing upper limb rehabilitation strategies. By tailoring interventions to target areas of maximal plastic potential, the SEI could serve as a key metric for guiding precision upper limb neurorehabilitation.

Future research should explore, in wider stroke populations, the relationship of SEI with lesion size, location, and chronicity to further evaluate its role as a diagnostic and prognostic tool. This aligns with the growing use of qEEG indices in neurology as neural markers for both clinical assessment and the development of novel neurorehabilitation approaches (Ahmadlou et al., 2010; Fasano et al., 2022; Geraedts et al., 2018; Lanzone et al., 2024).

By combining advanced neurophysiological metrics with innovative rehabilitation techniques such as VR and robotic exoskeletons, our study provides a robust framework for assessing and enhancing upper limb recovery in patients with stroke. The insights obtained from our pilot study indeed played a crucial role in shaping the neurophysiological assessment framework for a multicenter study using HD-EEG, with the aim of evaluating the spectral EEG modifications as a prognostic marker for recovery in a substantial cohort of patients with stroke (registered at ClinicalTrials.gov under identifier NCT06547827).

### 4.4 Limitations

The main limitation of this study is the relatively small sample size, which reduces statistical power and limits the generalizability of the findings (see Supplementary material for a *post-hoc* power analysis). The exploratory nature of the study means that SEI should be considered only as preliminary evidence of a potential biomarker of stroke recovery. Further, and adequately powered, studies on a larger population should be conducted to understand the sensitivity and specificity of SEI in stroke rehabilitation.

The relatively brief follow-up period may limit the interpretation of our findings at T2, and does not allow assessment of long-term neurophysiological and clinical effects. Further research with longer clinical and neurophysiological monitoring durations is needed to gain a comprehensive understanding of the interventions' long-term effects. Furthermore, since clinical scales were not administered at T2, it is not possible to determine whether there is a direct correlation between the neuroplastic changes observed at T2 and the clinical outcomes, although it is realistic to think that the upper limb clinical improvements obtained at T1 would also be confirmed at T2 [as the clinical data does not change after 1 week, as confirmed in our previous trial (Aprile et al., 2020)]. One possibility is that the neurophysiological changes induced by robotic rehabilitation with virtual reality—visible only after 30 therapy sessions—activate brain networks that drive functional improvements in the upper limbs, which are likely to be maintained for at least 3 months following treatment (as demonstrated by our previous studies). Therefore, the neurophysiological improvement of the SEI is transient, whereas the clinical-functional improvement in the upper limb is sustained. Further evaluations that combine both clinical assessments and neurophysiological data at later time points are necessary to better understand their relationship.

Given our small sample, we were underpowered to test dominance- or age-related interactions formally; larger studies will stratify by dominance/lesion side, adjust for age, and include lesion metrics to better account for these sources of variability.

We relied on a single qEEG index, the SEI, to assess localized cortical changes in our cohort. Incorporating additional EEG measures, such as those based on resting-state connectivity, could offer a more holistic understanding of brain dynamics.

## 5 Conclusion

This pilot randomized controlled trial provides preliminary evidence that the SEI can detect neurophysiological changes in the affected sensorimotor cortex of patients with subacute stroke undergoing VR-based robotic rehabilitation of the upper limb (mean difference of the SEI: T0 – T1 = −0.172, *p* = 0.037). These changes paralleled motor improvements but were not maintained at 1-week follow-up, and no differences emerged between unilateral and bilateral groups. Larger randomized trials with extended follow-up are needed to confirm these findings and clarify the clinical value of SEI as a biomarker of stroke recovery.

## Data Availability

The raw data supporting the conclusions of this article will be made available by the authors, without undue reservation.
