# Supplementary material for: Aperiodic brain activity changes in patients with stroke following virtual reality-based upper limb robotic rehabilitation: a pilot Randomized Controlled Trial
Source: Front Hum Neurosci. 2025 Oct 17;19:1671804. doi: 10.3389/fnhum.2025.1671804 (PMC12575235; doi:10.3389/fnhum.2025.1671804)
Supplement: Supplementary file 1 [file Data_Sheet_1.docx]

**Aperiodic brain activity changes in patients with stroke following virtual reality-based upper limb robotic rehabilitation: a pilot Randomized Controlled Trial**

**Maria Cristina Mauro****^†1*^, Alessio Fasano^†1*^, Marco Germanotta^1^, Laura Cortellini^1^, Sabina Insalaco^1^, Arianna Pavan^1^, Angela Comanducci^2^, Eugenio Guglielmelli^3^, Irene Giovanna Aprile^1^**

^1^ IRCCS Fondazione Don Carlo Gnocchi ONLUS, Florence, Italy

^2^ IRCCS Fondazione Don Carlo Gnocchi ONLUS, Milan, Italy

^3^ Department of Engineering, Università Campus Bio-Medico di Roma, Rome, Italy

†M.C. Mauro and A. Fasano are co-first authors.

*** Correspondence:**
Corresponding Authors

Maria Cristina Mauro (e-mail: mmauro@dongnocchi.it); Alessio Fasano, PhD (e-mail: [afasano@dongnocchi.it](mailto:afasano@dongnocchi.it))

# Supplementary S1 — Statistical power analyses for SEI outcomes

## Rationale

We quantified the statistical sensitivity of our finding on within-subject SEI (Time × Hemisphere in the sensorimotor cluster) and the ability to detect between-group differences (unilateral vs. bilateral) for our pilot RCT. Power analyses were performed with G*Power 3.1.9.7, using effect sizes and sphericity corrections estimated in SPSS from the present dataset, in order to keep the power computations anchored to the observed data structure. Greenhouse–Geisser (GG) procedures were applied because the Mauchly test indicated departure from sphericity for Time × Hemisphere.

## Data-anchored effect-size estimation

For the key within-subject effect (Time × Hemisphere), SPSS returned a partial η² of approximately 0.246 in the sensorimotor cluster, corresponding to Cohen’s f ≈ 0.57 via $f=\sqrt{\eta_{p}^{2}/(1-\eta_{p}^{2})}$*.* The Mauchly test’s results provided the GG-ε for this interaction (*ε*=0.626). Because repeated-measures power depends on the within-subject covariance, we computed the hemispheric contrast as affected – unaffected hemisphere (AH–UH) at each time point (T0, T1, T2), and estimated the average Pearson correlation among these three repeated contrast scores; the mean within-subject correlation was ≈0.48. For group-related tests we used the SPSS partial η² for the Time × Hemisphere × Group interaction (η²≈0.063, i.e., f≈0.26) together with the same ε and within-subject correlation.

## G*Power specification

For the within-subject effect, we used the “ANOVA: repeated measures, within factors” routine with three measurements (T0, T1, T2), n=18, α=0.05, f=0.57, average within-subject correlation ≈0.48, and GG-ε=0.626. For the between-group question, we used “ANOVA: repeated measures, within–between interaction” with two independent groups (unilateral, bilateral), three measurements, n=18 total, α=0.05, f≈0.26, average within-subject correlation ≈0.48, and GG-ε=0.626. We also ran an a-priori computation targeting 80% power for the same group-interaction settings to inform sample-size planning.

## Additional post-hoc analysis (independent-samples t-test on ΔSEI, UNI vs BI)

Because the primary between-group contrast in practice reduces to a difference in ΔSEI (T1–T0) across arms, we conducted a complementary post-hoc power analysis using a two-sample t-test. Group descriptives for the sensorimotor affected hemisphere were: UNI mean ΔSEI = 0.266 (SD = 0.342; n=9) and BI mean ΔSEI = 0.079 (SD = 0.137; n=9), yielding Cohen’s d = 0.718 (SD pooled ≈ 0.260). In G*Power we ran a t test → Means: Difference between two independent means, with two-tailed α = 0.05, n₁ = n₂ = 9, and d = 0.7178

## Results

The within-subject Time × Hemisphere effect in the sensorimotor cluster showed high achieved power (≈0.99) under the stated parameters, which is coherent with the large effect size and the significant modulation of SEI observed immediately post-treatment. In contrast, the group-related interaction exhibited low achieved power (≈0.49) at n=18, indicating that the null finding for unilateral vs. bilateral should be interpreted as inconclusive rather than as evidence of equivalence. The a-priori analysis suggested that a total sample of approximately 36 participants would be required to attain 80% power to detect a group interaction of the observed magnitude under the same sphericity and covariance assumptions.

The complementary post**-**hoc **independent-samples t-test** on ΔSEI returned an achieved power ≈ 0.30. This confirms that, for the observed between-group ΔSEI contrast, the present study was markedly underpowered.

## Interpretation and implications

These power estimates reconcile two aspects of our dataset. First, the within-subject aperiodic changes captured by the SEI in the affected sensorimotor cortex were large enough to be detected reliably in this pilot. Second, detecting modality-specific differences between unilateral and bilateral robotic training requires a substantially larger sample, given the smaller observed interaction effect. Consequently, future RCTs that aim to compare rehabilitation modalities on SEI-based endpoints should be powered at least to the order of ~36 analyzed participants under similar design parameters, with the understanding that deviations in sphericity or within-subject correlation will shift this requirement.

## Software and references

Analyses were performed in G*Power 3.1.9.7 and SPSS (version 28, IBM Corp., Armonk, NY, USA). The GG correction follows Greenhouse and Geisser (Psychometrika, 1959, 24:95–112). G*Power methodology is described in Faul et al. (Behav Res Methods, 2007, 39:175–191; 2009, 41:1149–1160).
